# Sex-related differences in risk factors, type of treatment received and outcomes in patients with atrial fibrillation and acute stroke: Results from the RAF-study (Early Recurrence and Cerebral Bleeding in Patients with Acute Ischemic Stroke and Atrial Fibrillation)

**DOI:** 10.1177/2396987316679577

**Published:** 2017-03-01

**Authors:** Kateryna Antonenko, Maurizio Paciaroni, Giancarlo Agnelli, Nicola Falocci, Cecilia Becattini, Simona Marcheselli, Christina Rueckert, Alessandro Pezzini, Loris Poli, Alessandro Padovani, Laszló Csiba, Lilla Szabó, Sung-Il Sohn, Tiziana Tassinari, Azmil H Abdul-Rahim, Patrik Michel, Maria Cordier, Peter Vanacker, Suzette Remillard, Andrea Alberti, Michele Venti, Monica Acciarresi, Cataldo D’Amore, Umberto Scoditti, Licia Denti, Giovanni Orlandi, Alberto Chiti, Gino Gialdini, Paolo Bovi, Monica Carletti, Alberto Rigatelli, Jukka Putaala, Turgut Tatlisumak, Luca Masotti, Gianni Lorenzini, Rossana Tassi, Francesca Guideri, Giuseppe Martini, Georgios Tsivgoulis, Kostantinos Vadikolias, Sokratis G Papageorgiou, Francesco Corea, Massimo Del Sette, Walter Ageno, Maria Luisa De Lodovici, Giorgio Bono, Antonio Baldi, Sebastiano D’Anna, Simona Sacco, Antonio Carolei, Cindy Tiseo, Davide Imberti, Dorjan Zabzuni, Boris Doronin, Vera Volodina, Domenico Consoli, Franco Galati, Alessio Pieroni, Danilo Toni, Serena Monaco, Mario M Baronello, Kristian Barlinn, Lars-Peder Pallesen, Jessica Kepplinger, Ulf Bodechtel, Johannes Gerber, Dirk Deleu, Gayane Melikyan, Faisal Ibrahim, Naveed Akhtar, Maria G Mosconi, Kennedy R Lees, Valeria Caso

**Affiliations:** 1 Department of Neurology, Bogomolets National Medical University, Kyiv, Ukraine; 2 Stroke Unit and Division of Cardiovascular Medicine, University of Perugia, Italy; 3 Neurologia d’urgenza e Stroke Unit, Istituto Clinico Humanitas, Rozzano, Milano, Italy; 4 Abteilung für Neurologie, Oberschwabenklinik gGmbH, Ravensburg, Germany; 5 Department of Clinical and Experimental Sciences, Neurology Unit, University “Health and Wealth” of Brescia, Italy; 6 Stroke Unit, University of Debrecen, Hungary; 7 Department of Neurology, Keimyung University School of Medicine, Daegu, South Korea; 8 Stroke Unit-Department of Neurology, Santa Corona Hospital, Pietra Ligure (Savona), Italy; 9 Medical School and Institute of Cardiovascular and Medical Sciences, University of Glasgow, Glasgow, United Kingdom; 10 Centre Cerebrovasculaire, Service de Neurologie, Department des Neurosciences Cliniques Centre Hopitalier Universitaire Vaudois, Lausanne, Switzerland; 11 Department of Neurology, Born Bunge Institute, Antwerp University Hospital, Antwerp, Belgium; 12 Stroke Unit, Neuroscience Department, University of Parma, Italy; 13 Stroke Unit, Dipartimento Geriatrico Riabilitativo, University of Parma, Italy; 14 Clinica Neurologica, Azienda Ospedaliero-Universitaria, Pisa, Italy; 15 SSO Stroke Unit, UO Neurologia, DAI di Neuroscienze, AOUI Verona, Italy; 16 Department of Neurology, Helsinki University Central Hospital, Helsinki, Finland; 17 Institute of Neuroscience and Physiology, Sahlgrenska Academy at University of Gothenburg, Gothenburg, Sweden; 18 Department of Neurology, Sahlgrenska University Hospital, Gothenburg, Sweden; 19 Department of Internal Medicine, Cecina Hospital, Cecina, Livorno, Italy; 20 Stroke Unit, AOU Senese, Siena, Italy; 21 Department of Neurology, Democritus University of Thrace, University Hospital of Alexandroupolis, Greece; 22 International Clinic Research Center, St. Anne’s University Hospital Brno, Brno, Czech Republic; 23 Second Department of Neurology, “Attikon” Hospital, University of Athens, School of Medicine, Athens, Greece; 24 UO Gravi Cerebrolesioni, San Giovanni Battista Hospital, Foligno, Italy; 25 Stroke Unit, Department of Neurology, Sant’Andrea Hospital, La Spezia, Italy; 26 Department of Internal Medicine, Insubria University, Varese, Italy; 27 Stroke Unit, Neurology, Insubria University, Varese, Italy; 28 Stroke Unit, Ospedale di Portogruaro, Portogruaro, Venice, Italy; 29 Department of Neurology, University of L’Aquila, Italy; 30 Department of Internal Medicine, Ospedale Civile di Piacenza, Italy; 31 Municipal Budgetary Healthcare Institution of Novosibirsk, City Clinical Hospital, Novosibirsk, Russia; 32 Stroke Unit, Jazzolino Hospital, Vibo Valentia, Italy; 33 Department of Neurology and Psychiatry, Sapienza University of Rome, Italy; 34 Stroke Unit, Ospedale Civico, Palermo; 35 Department of Neurology, Dresden University Stroke Center, Dresden, Germany; 36 Neurology, Hamad Medical Corporation, Doha, Qatar

**Keywords:** Sex differences, atrial fibrillation, ischemic stroke, secondary prevention, anticoagulation therapy, stroke outcome

## Abstract

**Introduction:**

Atrial fibrillation is an independent risk factor of thromboembolism. Women with atrial fibrillation are at a higher overall risk for stroke compared to men with atrial fibrillation. The aim of this study was to evaluate for sex differences in patients with acute stroke and atrial fibrillation, regarding risk factors, treatments received and outcomes.

**Methods:**

Data were analyzed from the “Recurrence and Cerebral Bleeding in Patients with Acute Ischemic Stroke and Atrial Fibrillation” (RAF-study), a prospective, multicenter, international study including only patients with acute stroke and atrial fibrillation. Patients were followed up for 90 days. Disability was measured by the modified Rankin Scale (0–2 favorable outcome, 3–6 unfavorable outcome).

**Results:**

Of the 1029 patients enrolled, 561 were women (54.5%) (*p* < 0.001) and younger (*p* < 0.001) compared to men. In patients with known atrial fibrillation, women were less likely to receive oral anticoagulants before index stroke (*p* = 0.026) and were less likely to receive anticoagulants after stroke (71.3% versus 78.4%, *p* = 0.01). There was no observed sex difference regarding the time of starting anticoagulant therapy between the two groups (6.4 ± 11.7 days for men versus 6.5 ± 12.4 days for women, *p* = 0.902). Men presented with more severe strokes at onset (mean NIHSS 9.2 ± 6.9 versus 8.1 ± 7.5, *p* < 0.001). Within 90 days, 46 (8.2%) recurrent ischemic events (stroke/TIA/systemic embolism) and 19 (3.4%) symptomatic cerebral bleedings were found in women compared to 30 (6.4%) and 18 (3.8%) in men (*p* = 0.28 and *p* = 0.74). At 90 days, 57.7% of women were disabled or deceased, compared to 41.1% of the men (*p* < 0.001). Multivariate analysis did not confirm this significance.

**Conclusions:**

Women with atrial fibrillation were less likely to receive oral anticoagulants prior to and after stroke compared to men with atrial fibrillation, and when stroke occurred, regardless of the fact that in our study women were younger and with less severe stroke, outcomes did not differ between the sexes.

## Introduction

Strokes in atrial fibrillation (AF) patients are common and frequently devastating (70–80% of patients die or become disabled,^1,2^ yet these strokes are preventable with anticoagulant therapy: 64% reduction in the risk of stroke and 25% reduction in mortality.^3^

Recent evidence shows that women are more frequently affected by AF compared to men, and have a higher associated risk for thromboembolic events.^4–6^ Therefore, female sex has been added as an independent risk-factor when calculating the CHA_2_DS_2_-VASc-score.^7^ Despite of this increased risk, women with AF still tend to be less treated with anticoagulants.^8,9^

The “Early Recurrence and Cerebral Bleeding in Patients with Acute Ischemic Stroke and Atrial Fibrillation” (RAF) Study investigated for (1) the risk of recurrent ischemic event and severe bleeding; (2) the risk factors for recurrence and bleeding; and (3) the risks of recurrence and bleeding associated with anticoagulant therapy and its starting time after the acute stroke.^10^ The results of this study have been recently published.^10^

The aim of this study was to evaluate the sex-differences in patients with acute stroke and AF, regarding risk factors, treatments received and outcomes.

## Methods

The methods and results of the RAF-study have been published recently.^10^ Briefly, RAF-study was performed between January 2012 and March 2014 and included 29 Stroke Units across Europe and Asia. All of the participating 29 Stroke Units provided standard stroke unit care and monitoring.^10^

On admission, stroke severity was assessed using the National Institutes of Health Stroke Scale (NIHSS). All patients underwent cerebral computed tomography examination without contrast or cerebral magnetic resonance to exclude intracranial hemorrhage. Thrombolysis treatment was administered as per local standard protocol, when appropriate. All patients were monitored for blood pressure, temperature, glucose level, heart rate, and blood gases in the first days after stroke. The choice of anticoagulant treatment (low molecular weight heparin [LMWH] or oral anticoagulants), as well as the day of its initiation, was left to the discretion of the treating physicians. AF was classified as paroxysmal, persistent, or permanent. A second brain computed tomography scan or magnetic resonance was performed 24–72 h from stroke onset in all patients. Hemorrhagic transformation was defined as any degree of hyperdensity within the area of low attenuation and was classified as either hemorrhagic infarction or parenchymal hematoma.^11,12^ The sites and sizes of the qualifying infarcts were determined based on standard templates as small, medium, large anterior or large posterior infarctions.^11,13,14^ Data on known stroke risk factors and treatment were collected and reported in the main paper.^10^ The CHA_2_DS_2_-VASc score before the index event was also calculated. The standard protocol also included a transthoracic echocardiography (TTE) during the hospital stay. Patients were followed up prospectively through face-to-face or telephone interviews. Study outcomes were (1) recurrent ischemic cerebrovascular events (stroke or TIA) and symptomatic systemic embolism; (2) symptomatic cerebral bleedings and major extracerebral bleeding at 90 days. The primary outcome was the composite of stroke, TIA, symptomatic systemic embolism, symptomatic cerebral bleeding, and major extracerebral bleeding. Disability and mortality at 90 days were also assessed using the modified Rankin Scale (mRS). Functional outcome was defined as either favorable (mRS 0–2) or unfavorable (mRS 3–6).

## Statistical analysis

Continuous variables, as well as NIHSS score, were reported as mean ± SD, and categorical variables were reported as percentages. Pearson’s chi-square test was used to compare categorized proportions. A comparison of discrete variables was conducted using a non-parametric test (Mann–Whitney). Multivariate logistic regression was performed in order to investigate sex differences for dichotomous outcomes. Included variables were: age, vascular risk factors, NIHSS on admission, the type of AF, lesion size, antithrombotic treatment before and after stroke, previous use of statins. The decision concerning which variables to include and to adjust in the multivariable analysis was guided by either the presence of an *a priori* theoretical or biological relationship among the examined patient characteristics as well as primary endpoints.^15^ A two-sided *p* < 0.05 was considered significant for all statistical tests. All statistical analyses were performed using software SPSS/PC Win package 20.0.

## Results

Overall, 1037 consecutive patients were enrolled in the study (59 from Asia) and 1029 were included in the analysis (eight excluded for incomplete data). Overall, 561 women were included (54.5%, *p* < 0.001), and they were on average younger (*p* < 0.001) compared to men. There was no observed difference in AF subtypes (paroxysmal, persistent or permanent) between the sexes (Table 1). History of myocardial infarction was more common in men (*p* = 0.002), as was history of peripheral arterial disease (*p* = 0.003) and aortic atherosclerosis (*p* = 0.016). Also, men more often had a pacemaker (*p* = 0.023) and were more often taking statins at the time of stroke onset (*p* < 0.001), while smoking and alcohol abuse were more common in men (*p* < 0.001). Previous use of antiplatelet agents was not significantly different between the two groups, while the use of anticoagulants was less frequent in women (25.5% versus 31.9%, respectively, *p* = 0.026).

**Table 1.** Baseline characteristics of patients.

Variables	Women	Men	*p*-Value
Age (years) (mean ± SD)	75.1 ± 9.04	77.2 ± 9.73	<0.001
Type of AF, *n* (%):			
Paroxysmal	198 (35.4)	166 (35.6)	0.948
Permanent	248 (44.4)	224 (48.1)	0.232
Persistent	115 (20.2)	76 (16.3)	0.091
Concomitant medical history, *n* (%):			
Hypertension	457 (82.3)	364 (78.3)	0.113
Hyperlipidemia	161 (29.2)	171 (36.9)	0.011
Diabetes mellitus	144 (25.9)	120 (25.8)	1.0
History of stroke/TIA	138 (25.0)	127 (27.4)	0.39
Chronic heart failure	99 (17.7)	94 (20.2)	0.336
History of myocardial infarction	72 (13.0)	94 (20.2)	0.002
History of peripheral artery disease	36 (6.49)	56 (12.1)	0.003
Aortic atherosclerosis	44 (7.86)	58 (12.5)	0.016
Atherosclerosis in other sites^a^	115 (21.3)	116 (25.2)	0.153
Pacemaker	36 (6.45)	49 (10.5)	0.023
Smoking	67 (12.1)	197(42.4)	<0.001
Alcoholism	7 (1.25)	61 (13.1)	<0.001
Previous use of oral anticoagulants, *n* (%):	141 (25.5)	148 (31.9)	0.026
Previous use of antiplatelets, *n* (%)	248 (44.8)	217 (46.9)	0.528
Previous use of statins, *n* (%)	115 (20.8)	145 (31.2)	<0.001
Lesion site and size, *n* (%):			
Small lesion	187 (33.4)	192 (41.1)	0.011
Medium lesion	202 (36.1)	166 (35.5)	0.896
Large anterior lesion	137 (24.5)	83 (17.8)	0.012
Large posterior lesion	34 (6.1)	25 (5.4)	0.687
Leukoaraiosis, *n* (%)	261 (47.1)	165 (35.9)	<0.001
NIHSS, mean ± SD	8.1 ± 7.5	9.2 ± 6.9	<0.001
Systolic AP, mean ± SD	148.4 ± 25.2	150.1 ± 25.3	0.048
Diastolic AP, mean ± SD	82.8 ± 14.3	82.9 ± 14.1	0.923
Laboratory data on admission, mean ± SD:			
Hemoglobin (g/dL), mean ± SD	14.3 ± 14.2	14.7 ± 12.3	0.65
Glycemia (mg/dL), mean ± SD	130.5 ± 39.8	223.5 ± 48.2	0.362
Total cholesterol (mg/dL), mean ± SD	171.8 ± 41.3	180.6 ± 42.8	<0.001
INR, mean ± SD	2.2 ± 3.3	3.5 ± 13.6	0.152

a Presence of internal carotid/vertebral artery stenosis ≥50%. AF: atrial fibrillation; AP: arterial pressure; INR: International Normalized Ratios at admission for all patients; TIA: transient ischemic attack.

Both large anterior lesions (*p* = 0.012) and leukoaraiosis (*p* < 0.001) were more frequent in women (Table 1). On transthoracic echocardiography, performed on 853 patients, mitral disease, aortic disease and severe atrial enlargement were more frequent in women (*p* = 0.025, *p* = 0.03 and *p* = 0.007, respectively), while cardiomyopathy was more frequent in men (*p* = 0.001). Tricuspid disease, and presence of aortic or mitral prostheses were not significantly different between the sexes (*p* = ns) (Table 2).

**Table 2.** Findings on transthoracic echocardiography, performed in 843 patients.

Variables	Women	Men	*p*-value
Atrial enlargement, *n* (%):	304 (68.5)	244 (62.7)	0.092
Mild	75 (28.3)	70 (26.8)	0.77
Moderate	113 (39.6)	90 (35.2)	0.287
Severe	116 (43.8)	81 (32.1)	0.007
Intracardiac thrombus, *n* (%)	4 (0.9)	7 (1.8)	0.383
Cardiomyopathy, *n* (%)	37 (8.31)	62 (15.9)	0.001
Mitral disease, *n* (%)	209 (46.8)	152 (39.0)	0.025
Aortic disease, *n* (%)	137 (30.6)	93 (23.9)	0.03
Tricuspid disease, *n* (%)	118 (26.3)	95 (24.5)	0.578
Biologic aortic prosthesis	4 (0.8)	6 (1.5)	0.526
Mechanical aortic prosthesis, *n* (%)	8 (1.7)	9 (2.2)	0.627
Biologic mitral prosthesis, *n* (%)	4 (0.8)	4 (0.7)	1.0
Mechanical mitral prosthesis, *n* (%)	15(3.2)	8 (2.0)	0.298

Types of revascularization therapy administered after ischemic stroke did not differ between the two groups. Anticoagulants were less often prescribed in women than in men after index stroke (71.3% versus 78.4%, respectively, *p* = 0.01). There were no sex differences regarding the time of initiating anticoagulant therapy between the two groups: (6.4 ± 11.7 days for men versus 6.5 ± 12.4 days for women, *p* = 0.902) (Table 3). A CHA_2_DS_2_-VASc score of 3 was found in 5.7% and 27.8% for women and men, respectively (*p* < 0.001), while a score between 7 and 9 was recorded more commonly in women (12.2% and 4.4% for women and men, respectively, *p* < 0.001). Men had more severe strokes than women on NIHSS (mean 9.2 ± 6.9 versus 8.1 ± 7.5, respectively, *p* < 0.001). Within 90 days, women had 46 (8.2%) recurrent ischemic events (stroke/TIA/systemic embolism) and 19 (3.4%) symptomatic cerebral bleedings compared to 30 (6.4%) and 18 (3.8%), respectively, in men (*p* = 0.28 and *p* = 0.74). At 90 days, 57.7% women were disabled or deceased compared to 41.1% of men (*p* < 0.001) (Table 3). In multivariate analysis, this significance was not confirmed (for unfavorable outcome – odds ratio (OR), 0.783, 95% confidence interval (CI), 0.536–1.143, *p* = 0.205 and for mortality – OR, 1.287, 95% CI, 0.726–2.284, *p* = 0.388) (Table 4).

**Table 3.** Treatment of patients in the acute period of stroke and outcome effects at 90 days.

Variables	Women	Men	*p*-Value
Revascularization therapy (IV and/or IA), *n* (%)	115 (20.6)	96 (20.6)	1.0
HT on neuroimaging^a^, *n* (%):	79 (14.1)	55 (11.9)	0.307
hemorrhagic infarction, *n* (%)	55 (11.0)	37 (9.1)	0.437
parenchymal hematoma, *n* (%)	24 (4.9)	18 (4.4)	0.874
Therapy with anticoagulants after index stroke, *n* (%)	399 (71.3)	367 (78.4)	0.01
Type of anticoagulation, *n* (%):			
LMWH	68 (12.1)	45 (9.6)	0.197
Oral anticoagulants (warfarin/DOA)	196 (35.0)	181 (38.7)	0.22
Bridging therapy (LMWH, followed by oral anticoagulants)	135 (24.1)	141 (30.1)	0.03
No anticoagulants at all	161 (28.8)	101 (21.6)	0.009
Time when anticoagulant therapy was initiated, mean ± SD, days	6.5 ± 12.4	6.4 ± 11.7	0.902
Outcome effects, *n* (%):			
Outcome ischemic events	46 (8.2)	30 (6.4)	0.284
Symptomatic HT	19 (3.4)	18 (3.8)	0.738
Mortality at 90 days	64 (11.6)	47 (10.1)	0.481
Unfavorable functional outcome (mRS 3-6) at 90 days	319 (57.7)	191 (41.1)	<0.001

a Neuroimaging performed after 24–72 h from stroke onset. DOA: direct oral anticoagulants; HT: hemorrhagic transformation (either hemorrhagic infarction or parenchymal hematoma); IA: intra-arterial revascularization therapy; IV: intravenous revascularization therapy; LMWH: low molecular weight heparin.

**Table 4.** Multivariate logistic regression model for dichotomous outcomes.

Variables	Unfavorable outcome			Mortality		
	OR	95% CI	p	OR	95% CI	P
Age	1.039	1.018–1.062	<0.001	1.044	1.009–1.079	0.014
NIHSS on admission	1.178	1.136–1.222	<0.001	1.076	1.037–1.118	<0.001
Diabetes mellitus	1.133	0.734–1.747	0.574	1.277	0.695–2.345	0.431
Previous use of antiplatelets	0.999	0.669–1.492	0.996	0.907	0.496–1.661	0.752
Previous use of oral anticoagulants	1.139	0.708–1.834	0.591	1.484	0.720–3.058	0.285
Previous use of statins	0.629	0.360–1.099	0.104	1.080	0.475–2.456	0.854
Hypertension	0.830	0.513–1.343	0.448	0.874	0.421–1.814	0.717
Hyperlipidemia	0.890	0.537–1.476	0.653	0.814	0.376–1.763	0.602
Paroxysmal AF	0.848	0.524–1.374	0.503	1.037	0.501–2.147	0.922
History of stroke/TIA	1.734	1.148–2.620	0.009	0.741	0.405–1.357	0.332
Smoking	0.939	0.673–1.308	0.709	1.318	0.833–2.086	0.238
Alcoholism	1.528	0.720–3.243	0.269	1.928	0.690–5.387	0.211
Chronic heart failure	1.235	0.767–1.989	0.385	0.962	0.501–1.845	0.907
History of myocardial infarction	1.194	0.737–1.934	0.470	1.240	0.638–2.411	0.526
Pacemaker	1.048	0.562–1.955	0.883	2.130	0.959–4.733	0.063
Small ischemic lesion	0.408	0.195–0.853	0.017	0.090	0.027–0.295	<0.001
Leukoaraiosis	0.991	0.688–1.427	0.961	1.394	0.816–2.379	0.224
Therapy with anticoagulants after index stroke	0.389	0.249–0.605	<0.001	0.237	0.137–0.411	<0.001

AF: atrial fibrillation; AP: arterial pressure; INR: International Normalized Ratios at admission for all patients; TIA: transient ischemic attack.

Regarding sex differences, East European Stroke Units had 71 patients: 33 women (46.5%) and 38 men (53.5%)) and other Stroke Units in Europe (900 patients: 492 women (54.7%) and 408 men (45.3%)). Comparing women and men from Eastern Europe, the former were older (mean age 79.9 ± 8.3 versus 71.2 ±11.8, respectively, *p* < 0.001) and had more severe strokes than men (mean NIHSS 10.0 ± 7.2 versus 5.8 ± 4.96, respectively, *p* = 0.006) (Supplementary Table 1). No differences in the rates of prescribing anticoagulants between women and men in East Europe before and after index stroke were observed. While, after index stroke, LMWHs were prescribed more often to women than men (66.7% versus 31.6%, *p* = 0.004) and much more often than oral anticoagulants in East Europe. There were no observed sex differences regarding the time of initiating anticoagulant therapy in East Europe: (3.95 ± 15.9 days for men versus 3.67 ± 7.5 days for women, *p* = 0.923). (Supplementary Table 2).

## Discussion

This study found that women overall in Europe with AF were less likely to receive oral anticoagulants prior to and after stroke compared to men and regardless of the fact that women were significantly younger and with less severe stroke at onset; outcomes did not differ between the sexes. The RAF-study included more women (54.5%) than men, who were on average younger and had less severe stroke at onset compared to previous studies.^16,17^ Moreover, while previous studies had included patients with all types of stroke and with and without AF,^16–18^ the RAF-study included only ischemic stroke patients with AF. Furthermore, women more frequently had mitral disease and severe atrial enlargement. The latter fact has been reported by Gómez-Doblas, who has stating that women have more rheumatic aetiologies, while men tend to be more affected by ischemic or congenital aetiologies.^19^ Rheumatic aetiologies have been correlated with a higher embolic risk of AF and an earlier onset of stroke.^20^

In this study, women were less likely to receive oral anticoagulants before the index stroke, a finding in line with past population studies.^8,9^ Likewise, women were less likely to receive anticoagulation therapy for secondary prevention. This clear disparity in treatment delivery has also been documented by the Austrian Stroke Unit Registry.^18^ This under-treatment for women has been hypothesized as being due to a lack of social support, as well as other concomitant diseases afflicting these patients, including cognitive decline, a higher burden of vascular brain disease, epilepsy and an increased risk of falls.^18^ Furthermore, we also observed that women had a lower rate of statin use, despite an equal rate of atherosclerosis between the sexes.^18,21^

Even though men were older and had more severe stroke, the mortality and disability rates between the sexes were similar. The selection of patient cohorts could have influenced this, as only cardioembolic strokes were included, which could also explain the younger age of women at stroke onset.

## Study limitations

This hospital-based clinical study was not randomized but based upon consecutively admitted patients fulfilling inclusion criteria.

## Conclusions

The RAF-study observed that women regardless of lower NIHSS-score at admission and younger age, experienced the same outcomes as men with higher NIHSS at admission and older age. Moreover, this study also observed that women were less likely, compared to men, to have been prescribed anticoagulants before and after stroke.

## Supplementary Material

Supplementary material
