# Supplementary material for: Sex-related differences in risk factors, type of treatment received and outcomes in patients with atrial fibrillation and acute stroke: Results from the RAF-study (Early Recurrence and Cerebral Bleeding in Patients with Acute Ischemic Stroke and Atrial Fibrillation)
Source: Eur Stroke J. 2017 Mar 1;2(1):46–53. doi: 10.1177/2396987316679577 (PMC6377059; doi:10.1177/2396987316679577)
Supplement: Supplementary material [file ESO_679577_Supplemental_Material.pdf]

Supplemental Table 1. Baseline characteristics of patients in East Europe and other parts of Europe.

Variables	EAST EUROPE			WEST, NORTH, SOUTH EUROPE		
	Women	Men	p-value	Women	Men	p-value
Age (years) (mean±SD)	79.9±8.3	71.2±11.8	<0.001	75.7±9.24	79.4±8.6	<0.001
Type of AF, n (%):						
Paroxysmal	8 (24.2)	10 (27.0)	1.0	190 (38.8)	152 (37.3)	0.679
Permanent	23 (69.7)	25 (67.6)	1.0	204 (41.6)	187 (46.1)	0.199
Persistent	1 (3.0)	2 (5.4)	1.0	99 (20.2)	68 (16.7)	0.197
Concomitant medical history, n (%):						
Hypertension	32 (97.0)	33 (89.2)	0.361	401 (82.5)	320 (78.8)	0.172
Hyperlipidemia	10 (32.3)	14 (37.8)	0.799	150 (30.9)	155 (38.3)	0.023
Diabetes mellitus	13 (39.4)	7 (18.9)	0.069	123 (25.3)	113 (27.8)	0.402
History of stroke/TIA	13 (39.4)	11 (29.7)	0.455	118 (24.4)	113 (27.9)	0.25
Chronic hearth failure	12 (36.4)	15 (40.5)	0.808	69 (14.1)	69 (17.0)	0.228
History of myocardial infarction	3 (9.1)	6 (16.2)	0.485	65 (13.4)	88 (21.7)	0.001
History of peripheral artery disease	4 (12.1)	12 (32.4)	0.051	32 (6.6)	44 (10.8)	0.03
Aortic atherosclerosis	4 (12.1)	7 (18.9)	0.522	40 (8.1)	51 (12.6)	0.034
Atherosclerosis* in other sites	17 (56.7)	24 (66.7)	0.452	97 (20.3)	88 (21.9)	0.618
Pacemaker	4 (12.1)	8 (21.6)	0.353	31 (6.3)	38 (9.4)	0.102
Smoking	0	13 (35.1)	<0.001	65 (13.4)	168 (41.4)	<0.001
Alcoholism	0	10 (27)	0.001	6 (1.2%)	34 (8.4)	<0.001
Previous use of oral anticoagulants, n (%):	13 (41.9)	18 (48.6)	0.631	116 (23.9)	126 (31.0)	0.019
Previous use of antiplatelets, n (%)	16 (51.6)	18 (48.6)	1.0	221 (45.5)	186 (45.9)	0.893
Previous use of statins, n (%)	7 (22.6)	13 (35.1)	0.296	104 (21.4)	129 (31.8)	0.001
Lesion site and size, n (%):						
Small lesion	15 (45.5)	19 (50.0)	0.813	161 (32.8)	163 (40.0)	0.026
Medium lesion	13 (39.4)	16 (42.1)	1.0	177 (36.0)	143 (35.1)	0.78
Large anterior lesion	2 (6.1)	0	0.212	122 (24.8)	78 (19.2)	0.044
Large posterior lesion	34 (6.1)	25 (5.4)	0.687	29 (5.9)	24 (5.9)	1.0
Leukoaraiosis, n (%)	21 (63.6)	18 (48.6)	0.236	231 (47.6)	147 (36.6)	0.001
NIHSS, mean±SD	10.0±7.2	5.8±4.96	0.006	8.5±6.0	10.2±7.4	0.001
Systolic AP, mean±SD	161.8±23.2	148.8±29.8	0.04	151.6±25.4	149.0±24.9	0.126
Diastolic AP, mean±SD	89.6±20.3	87.3±13.6	0.58	82.4±13.9	82.3±13.9	0.874
Laboratory data on admission, mean±SD:						
Hemoglobin (g/dL), mean±SD	12.8±1.44	14.7±1.9	<0.001	12.9±3.4	13.6±1.8	<0.001
Glycemia (mg/dL), mean±SD	137.0±47.7	116.7±37.6	0.056	130.4±47.6	132.4±49.2	0.543
Total cholesterol (mg/dL), mean±SD	190.5±45.4	172.1±37.8	0.116	171.9±41.2	188.2±41.1	<0.001
INR, mean±SD	1.25±0.56	1.5±0.86	0.098	1.26±0.77	1.26±0.46	0.963

*Presence of internal carotid/vertebral artery stenosis ≥50%
AF=atrial fibrillation
AP=arterial pressure
INR= International Normalized Ratios at admission for all patients
TIA=transient ischemic attack

Supplemental Table 2. Treatment of patients in the acute period of stroke and outcome effects at 90 days in patients of East Europe and other parts of Europe.

	EAST			WEST, NORTH, SOUTH EUROPE		
Variables	Women	Men	p-	Women	Men	p-value

			value			
Revascularization therapy (IV and/or IA), n (%)	7 (21.2)	6 (16.2)	0.76	97 (19.8)	84 (20.7)	0.739
HT on neuroimaging*, n (%):	0	2 (5.4)	0.494	67 (13.6)	52 (12.8)	0.767
hemorrhagic infarction, n (%)	0	1 (2.7)	1.0	47 (11.0)	36 (10.4)	0.817
parenchymal hematoma, n (%)	0	1 (2.7)	1.0	19 (4.5)	16 (4.6)	1.0
Therapy with anticoagulants after index stroke, n (%)	32 (97.0)	35 (92.1)	0.618	344 (70.1)	317 (77.7)	0.01
Type of anticoagulation, n (%):						
LMWH	22 (66.7)	12 (31.6)	0.004	45 (9.2)	32 (7.8)	0.48
Oral anticoagulants (warfarin/DOA)	4 (12.1)	7 (18.4)	0.527	179 (36.5)	172 (42.2)	0.08
Bridging therapy (LMWH, followed by oral anticoagulants)	6 (18.2)	16 (42.1)	0.04	120 (24.4)	113 (27.7)	0.267
No anticoagulants at all	1 (3.0)	3 (7.9)	0.618	147 (29.9)	91 (22.3)	0.01
Time when anticoagulant therapy was initiated, mean±SD, days	3.67±7.5	3.95±15.9	0.923	6.52±12.3	6.88±11.5	0.65
Outcome effects, n (%):						
Outcome ischemic events	4 (12.1)	2 (5.3)	0.406	39 (7.9)	26 (6.4)	0.438
Symptomatic HT	0	3 (7.9)	0.243	18 (3.7)	14 (3.4)	1.0
Mortality at 90 days	0	1 (2.6)	1.0	62 (12.7)	45 (11.1)	0.47
Unfavorable functional outcome (mRS 3-6) at 90 days	16 (48.5)	10 (26.3)	0.083	285 (58.5)	171 (42.1)	<0.001

*Neuroimaging performed after 24-72 hours from stroke onset
DOA=direct oral anticoagulants
HT=hemorrhagic transformation (either hemorrhagic infarction or parenchymal hematoma)
IA=intra-arterial revascularization therapy
IV=intravenous revascularization therapy
LMWH=low molecular weight heparin
